# Association of Psychosocial Conditions, Oral Health, and Dietary Variety with Intellectual Activity in Older Community-Dwelling Japanese Adults

**DOI:** 10.1371/journal.pone.0137656

**Published:** 2015-09-11

**Authors:** Kimiko Tomioka, Nozomi Okamoto, Norio Kurumatani, Hiroshi Hosoi

**Affiliations:** 1 Nara Prefectural Health Research Center, Nara Medical University, Kashihara, Nara, Japan; 2 Department of Community Health and Epidemiology, Nara Medical University, Kashihara, Nara, Japan; Hamamatsu University School of Medicine, JAPAN

## Abstract

**Background:**

This study examined the factors related to intellectual activity in community-dwelling elderly persons.

**Methods:**

Self-administered questionnaires mailed to all people aged ≥65 years in a dormitory suburb in Japan (n = 15,210). The response rate was 72.2%. Analytical subjects (n = 8,910) were those who lived independently and completely answered questions about independent and dependent variables and covariates. Independent variables included psychosocial conditions (i.e., social activities, hobbies, and a sense that life is worth living (*ikigai*)), oral health (i.e., dental health behaviors and oral function evaluated by chewing difficulties, swallowing difficulties, and oral dryness), and dietary variety measured using the dietary variety score (DVS). A dependent variable was intellectual activity measured using the Tokyo Metropolitan Institute of Gerontology Index of Competence. Covariates included age, gender, family structure, pensions, body mass index, alcohol, smoking, medical history, self-rated health, medications, cognitive function, depression, and falling. Logistic regression was used to estimate the odds ratio (OR) for poor intellectual activity.

**Results:**

Poor intellectual activity was reported by 28.9% of the study population. After adjustment for covariates and independent variables, poor intellectual activity was significantly associated with nonparticipation in social activities (OR = 1.90, 95%CI = 1.61–2.24), having neither hobbies nor *ikigai* (3.13, 2.55–3.84), having neither regular dental visits nor daily brushing (1.70, 1.35–2.14), the poorest oral function (1.61, 1.31–1.98), and the lowest DVS quartile (1.96, 1.70–2.26).

**Conclusion:**

These results indicate that psychosocial conditions, oral health, and dietary variety are independently associated with intellectual activity in elderly persons. The factors identified in this study may be used in community health programs for maintaining the intellectual activity ability of the elderly.

## Introduction

Functional decline in older adults is a serious problem in countries with increasingly aging populations, because it has an enormous effect on hospitalization, institutionalization, and death and leads to burgeoning cost of nursing care and medical treatment [1,2]. Therefore, it is imperative to identify the modifiable risk factors for functional decline in elderly adults at an early stage.

As for the concept of the hierarchical model of functional and behavioral competence, Lawton established a model of competence comprising seven stages in ascending order of complexity: life maintenance, functional health, perception-cognition, physical self-maintenance, instrumental self-maintenance, effectance, and social role [3]. Of these stages, complex abilities to cover the last three stages—instrumental self-maintenance (corresponding to instrumental activities of daily living; IADL), effectance (corresponding to intellectual activity), and social role performance—are important for an individual to live a socially independent life, and have been defined as higher-level functional capacity. Among the three stages of higher-level functional capacity, intellectual activity, which represents the motivation behind human needs to create tension, explore, and vary the psycho-environmental field, such as having an interest in health-related information through the mass media [3], has been recognized as an essential ingredient for healthy aging because good intellectual activity can decrease the risk of incident disability in IADL and cognitive decline among community-dwelling older adults [4–6].

Studies of community-dwelling elderly persons found that intellectual activity was associated with dental health behavior [7], self-perceived chewing ability [8], and dietary variety [9]. It has been further reported that self-perceived chewing ability is important for the maintenance of good dietary habits in the elderly [10]. Moreover, functional capacity of the elderly is considered to be affected by psychosocial conditions, such as participation in social activities, hobbies, and a sense that life is worth living (*ikigai*) [4,11,12], and a recent study has demonstrated a positive association between participation in social activities and oral health status [13]. These prior studies have suggested that psychosocial conditions, oral health, and dietary intake have a significant association with intellectual activity, and may well interrelate with each other, a possibility that has yet to be studied. Clarifying the comprehensive relationship between psychosocial conditions, oral health, dietary intake, and intellectual activity can help provide a better understanding for increasing the healthy life years of the elderly.

The purpose of the present study was to investigate the cross-sectional association of psychosocial conditions, oral health, and dietary intake with intellectual activity in an elderly, community-dwelling, self-independent adult population. We hypothesized that poor intellectual activity would be associated with poor psychosocial conditions, poor oral health, and poor dietary intake, and that these associations would be explained independently from risk factors for higher-level functional capacity, such as older age, poor socioeconomic status, poor lifestyle, chronic medical conditions, poor self-rated health, poor cognitive function, and depressive symptomatology [14–17], and higher-level functional capacity other than intellectual activity (i.e., IADL and social role).

## Methods

### Study Area and Subjects

**Fig 1:**
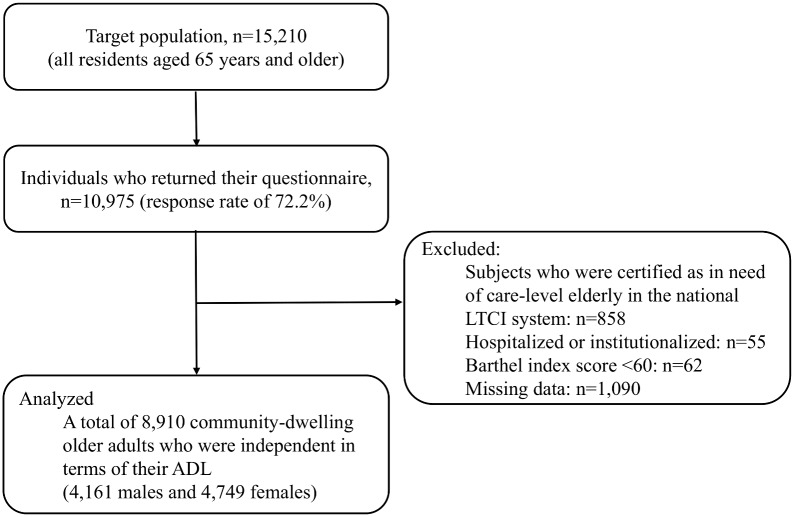
Selection of subjects. ADL, activities of daily living; LTCI, long-term care insurance.

The target area for this study was Kashiba City, located in the mid-west part of Nara prefecture. This city is a typical commuter town of the megacity, Osaka. At the end of January, 2014, the rate of the aging population was 19.9%. In March 2014, the city office distributed self-administered postal questionnaires to all community-dwelling individuals aged 65 years and older (n = 15,210). When the residents could not answer questions independently, a family member was asked to assist them or answer the questions on their behalf. Fig 1 shows the procedure for selecting subjects. Replies about the outcome were obtained from 10,975 residents (response rate, 72.2%). Among them, 975 individuals who had already experienced a dependency in ADL at the time of survey (858 subjects who were certified as in need of care-level elderly in the national long-term care insurance system, 55 subjects who were hospitalized or institutionalized, and 62 subjects who had severe functional dependency identified by using the Barthel index; scores of <60 [18]) were excluded because ADL sometimes has an influence on higher-level functional capacity. Additionally, 1,090 individuals were excluded due to missing data for covariates, psychosocial conditions, oral health, and dietary variety. Therefore, our analysis was performed with data from 8,910 community-dwelling older adults who were independent in terms of their ADL (age range 65–100, median 72, and interquartile range 68–77).

All study participants provided signed informed consent. This study protocol was approved by the Nara Medical University Ethics Committee (approval number 939).

### Assessment of Higher-Level Functional Capacity

Higher-level functional capacity was assessed using the Tokyo Metropolitan Institute of Gerontology Index of Competence (TMIG-IC) [19]. The TMIG-IC was developed to measure the competence required for community-residing older adults to live independently in the community. The TMIG-IC is a widely used standard in Japan, and its reliability and validity have been tested [20]. This is a multidimensional 13-item index (see S1 Table). The first five questions inquire about IADL, the subsequent four about intellectual activity, and the final four about social role. The respondent selects either "yes" (one point) or "no" (zero points). A higher score indicates a higher level of competence. Poor capacity in each subscale of the TMIG-IC was defined as a score of 1 or more below the respective full mark; below 5 for IADL and below 4 for intellectual activity and social role [6,7].

### Assessment of Social Activities

We evaluated the frequency of five types of social activities: volunteer groups, sports groups, senior citizens’ clubs, neighborhood community associations, and cultural clubs. Participants rated how often they participated in each activity based on a 5-point scale (0, never; 1, several times a year; 2, several times a month; 3, once a week; 4, several times a week; 5, four or more times a week). A sum score of social activities (minimum = 0, maximum = 25) was constructed by adding the frequency score (0–5) in each activity, which was defined as social activity score [21,22]. The participants were classified into quartiles according to their social activity score; a high frequency group (a score of ≥6), a medium frequency group (a score of 3–5), a low frequency group (a score of 1–2), and a non-participation group (a score of 0).

### Assessment of Hobbies and Life Worth Living (*Ikigai*)

Hobbies and life worth living (*ikigai*) were each assessed by asking a single question: “Do you have any hobbies?” and “Do you have *ikigai*?” which could be translated directly as “Do you have anything to live for?”. *Ikigai* refers to aspects of one's life that make it meaningful or worth living [12]. The response to each question was simply ‘yes’ or ‘no’. Subjects who answered ‘yes’ to the question were defined as persons who have hobbies or life worth living (*ikigai*).

### Assessment of Oral Health

In our study, oral health consisted of two parts of dental health behaviors and oral function. For assessment of dental health behaviors, regular visits to a dentist (yes or no), and brushing frequency (less than once a day or once a day or more) were selected because they are considered important dental health behaviors in older adults [7]. For assessment of oral function, three questionnaire items were used (Do you have difficulty chewing hard foods? Do you choke on your tea and soup? Do you have dry mouth?). These three variables are often considered relevant correlates of oral health and have been used within previous studies as measures of subjective oral health status [8,23,24]. The total number of negative answer to respective questions was tallied and participants were categorized as 0 (no oral dysfunction), 1, 2 or 3 (complaining of all three).

### Assessment of Dietary Intake

We used dietary variety as the indicator of dietary intake because it has been reported to be a straightforward tool for screening and identifying the elderly at nutritional risk [25]. Dietary variety was measured using the dietary variety score (DVS) [9]. DVS was assessed by a questionnaire of food frequency during 1 week, which covers the 10 main food groups in Japanese meals (meat, eggs, fish and shellfish, milk, dark-colored vegetables, soybean products, potatoes, fruits, seaweeds, and fats and oils). The response to each food group was either ‘have eaten almost every day (a score of 1)’, ‘not eaten for 1 day or more (a score of 0)’. DVS was designated as the sum of the 10 food groups, such that a higher score (maximum of 10 points) would indicate a higher level of dietary variety. The participants were classified into quartiles according to their DVS; highest DVS group (Q4, a score of 9–10), second highest DVS group (Q3, a score of 7–8), second lowest DVS group (Q2, a score of 5–6), and lowest DVS group (Q1, a score of 4 or less).

### Covariates

Covariates included demographic characteristics, such as age, gender, family structure, pensions, and body mass index (BMI); lifestyle habits, such as alcohol intake and history of smoking; and physical and mental status, such as medical history, self-rated health, the number of medications used, cognitive function, depressive symptoms, and falling.

Age groups were categorized as 65–69, 70–74, 75–79, 80–84, and ≥85 years. Family structure was categorized as living alone, living only with one’s spouse, living with a person other than spouse, and living with three or more people. Pensions were used as an indicator of socioeconomic status. Pensions were categorized as national pension, employees' pension, mutual aid association pension, and other (e.g., disability or survivor pension). BMI was categorized as underweight (i.e., <18.5), normal (i.e., 18.5-<25.0), and overweight (i.e., ≥25.0). Lifestyle habits asked about included alcohol intake (nondrinkers, social drinkers, occasional drinkers, or daily drinkers) and smoking history (never-smokers, ex-smokers, or current smokers). For medical history, self-reported histories of hypertension, cerebrovascular disease, heart disease, diabetes mellitus, chronic respiratory disease, musculoskeletal disorder, ophthalmologic disease, otological disease, and cancer of any kind was collected. Self-rated health was categorized as very good, good, fair, or poor. The number of medications used was categorized as none, 1–2, 3–4, or more than 5. Cognitive function was evaluated using the Cognitive Performance Scale (CPS: score range 0–6) [26], and a score ≥1 was considered indicative of the presence of poor cognitive functioning. Depression was evaluated using the 5-item short form of the Geriatric Depression Scale (GDS-5: score range 0–5) [27], and a score ≥2 was defined as the presence of depression. Falling was measured by the question, “Have you fallen in the past year?” to which respondents answered “yes” or “no”.

### Statistical Analysis

Study variables’ differences between genders were analyzed using Fisher's exact test. The trend test was performed using the Cochran-Armitage test. Multiple logistic regression analysis (by the forced entry method) was carried out using “with or without poor intellectual activity (intellectual activity score <4 or full mark)” as a dependent variable. Independent variables were psychosocial conditions (i.e., social activities, hobbies, and life worth living), oral health (i.e., dental health behaviors and oral function), and dietary variety. Age (dummy variable with the aged 65–69 group as a reference), gender (dummy variable with males as a reference), family structure (dummy variable with the living with three or more people group as a reference), pensions (dummy variable with the national pension group as a reference), BMI (dummy variable with the normal group as a reference), alcohol intake (dummy variable with the nondrinkers as a reference), smoking history (dummy variable with the never-smokers as a reference), medical history (dummy variable with the subjects without a disease as a reference), self-rated health (dummy variable with the very good group as a reference), the number of medications used (dummy variable with the none group as a reference), cognitive function (dummy variable with the subjects without poor cognitive functioning as a reference), depression (dummy variable with the subjects without depression as a reference), and experiences with falling (dummy variable with the no falling group as a reference) were included as covariates. The results were shown as odds ratio (OR) with 95% confidence interval (CI). We first adjusted for covariates (Model 1); the association of psychosocial conditions, oral health, and dietary variety on poor intellectual activity was examined separately. Subsequently, in addition to Model 1, psychosocial conditions, oral health, and dietary variety were included in the model simultaneously (Model 2). Finally, IADL was added to the variables in Model 2 (Model 3), and social role was added to the variables in Model 2 (Model 4). For the association of frequency of participation in social activities, the number of impaired oral function, and dietary variety with poor intellectual activity, a linear trend test was also conducted to assess its dose—response relationship. The level of significance was 0.05 (two tailed). Statistical analyses were performed using SPSS (version 17.0; SPSS Japan Inc., Tokyo, Japan).

### Results

Of the analytical subjects, 71.1% reported having independent intellectual activity and 28.9% reported having poor intellectual activity. Table 1 shows the psychosocial conditions, oral health variables, dietary variety score, and higher-level functional capacity among study participants according to gender. Males significantly had more hobbies, poorer dental health behaviors, less dietary variety, lower IADL, and lower social role than females. There were no differences in the percentage of psychosocial conditions except hobbies, oral function variables, and intellectual activity between males and females.

**Table 1:** Psychosocial Conditions, Oral Health, Dietary Variety, and Higher-level Functional Capacity among Study Participants according to Gender.

Characteristics	All	Males	Females	P^a^
	n = 8,910	n = 4,161	n = 4,749	
**Psychosocial conditions**				
Having no hobbies	15.6%	14.6%	16.6%	0.010
Lack of life worth living (no *ikigai*)	12.2%	12.4%	12.1%	0.650
No participation in social activities	37.9%	36.9%	38.8%	0.070
**Oral health variables**				
Lack of daily brushing	7.7%	9.3%	6.4%	<0.001
Lack of regular dental visits	50.8%	53.2%	48.7%	<0.001
Having difficulty in chewing hard foods	31.5%	32.1%	31.0%	0.280
Choking on your tea and soup	24.2%	23.5%	24.8%	0.160
Having dry mouth	26.0%	26.0%	26.0%	1.000
**Dietary variety score (DVS)**				
Quartile 4 (DVS ≥9)	29.5%	23.5%	34.8%	<0.001
Quartile 3 (DVS = 7–8)	23.0%	20.3%	25.4%	
Quartile 2 (DVS = 5–6)	20.4%	21.1%	19.8%	
Quartile 1 (DVS ≤4)	27.1%	35.1%	20.0%	
**Higher-level functional capacity**				
Poor IADL (≤4)	12.4%	17.1%	8.2%	<0.001
Poor intellectual activity (≤3)	28.9%	29.8%	28.1%	0.080
Poor social role (≤3)	46.7%	53.3%	40.9%	<0.001

IADL, Instrumental activities of daily living.

^a^ Differences between gender were analyzed using Fisher's exact test.

Tables 2 and 3 shows the characteristics of the subjects stratified by the level of intellectual activity. The factors showing a significant dose-response relationship in the trend test were age, living alone, national pension, being underweight, not drinking, currently smoking, a medical history of cerebrovascular disease, heart disease, chronic respiratory disease, musculoskeletal disorder, otological disease, and ophthalmologic disease, bad self-rated health, the number of medications used, poor cognitive functioning, depressive symptoms, experience with falling, all of the psychosocial conditions, all of the oral health variables, low dietary variety, poor IADL, and poor social role.

**Table 2:** Demographics, Lifestyle Habits, and Physical and Mental Status of Participants Stratified by the Level of Intellectual Activity.

Characteristics	High IA	Medium IA	Low IA	P^a^
	n = 6,336	n = 1,516	n = 1,058	
**Demographics**				
Male	46.1%	49.7%	46.0%	0.150
Age ≥75 years	34.2%	38.0%	54.0%	<0.001
Living alone	9.9%	11.7%	15.5%	<0.001
National pension	33.2%	35.5%	43.3%	<0.001
Underweight (BMI<18.5)	6.3%	7.8%	7.6%	0.020
**Lifestyle habits**				
Non-drinkers	35.5%	38.1%	47.4%	<0.001
Current smokers	8.1%	12.3%	14.3%	<0.001
**Physical and mental conditions**				
Hypertension	38.2%	39.0%	41.0%	0.110
Cerebrovascular disease	3.0%	4.4%	5.4%	<0.001
Heart disease	9.8%	12.4%	11.7%	0.002
Diabetes mellitus	11.8%	11.2%	14.3%	0.220
Chronic respiratory disease	5.0%	5.7%	6.5%	0.030
Musculoskeletal disorder	14.0%	14.2%	18.9%	0.002
Otological disease	17.2%	19.1%	21.2%	0.001
Ophthalmologic disease	6.1%	8.8%	8.2%	<0.001
Cancer	3.4%	3.8%	4.3%	0.150
Self-rated health (fair or poor)	17.1%	24.8%	38.9%	<0.001
The number of medications used (≥5)	20.6%	27.7%	35.7%	<0.001
Poor cognitive functioning (CPS≥1)	12.6%	24.7%	40.3%	<0.001
Depressive symptoms (GDS5≥2)	18.3%	29.7%	45.0%	<0.001
Experience with falling in the past year	16.7%	20.5%	34.3%	<0.001

BMI, body mass index; CPS, Cognitive Performance Scale; GDS, Geriatric Depression Scale; IA, intellectual activity.

High IA means a score of 4. Medium IA means a score of 3. Low IA means a score of 2 or less.

^a^ P for trend by the Cochran-Armitage test.

**Table 3:** Psychosocial Conditions, Oral Health, Dietary Variety, and Higher-level Functional Capacity of Participants Stratified by the Level of Intellectual Activity.

Characteristics	High IA	Medium IA	Low IA	P^a^
	n = 6,336	n = 1,516	n = 1,058	
**Psychosocial conditions**				
No participation in social activities	32.1%	44.9%	62.1%	<0.001
Having no hobbies	8.8%	23.5%	45.2%	<0.001
Lack of life worth living (no *ikigai*)	7.6%	17.2%	32.6%	<0.001
**Oral health variables**				
Lack of daily brushing	6.7%	8.4%	12.9%	<0.001
Lack of regular dental visits	46.3%	59.6%	65.1%	<0.001
Having difficulty in chewing hard foods	26.7%	37.1%	52.3%	<0.001
Choking on your tea and soup	21.0%	28.1%	37.8%	<0.001
Having dry mouth	22.9%	29.9%	38.9%	<0.001
**Dietary variety**				
Low variety (Dietary variety score ≤6)	42.4%	55.9%	65.6%	<0.001
**Higher-level functional capacity**				
Poor IADL (score ≤4)	7.0%	15.5%	40.2%	<0.001
Poor social role (score ≤3)	37.4%	62.6%	79.6%	<0.001

IADL, instrumental activities of daily living.

High IA means a score of 4. Medium IA means a score of 3. Low IA means a score of 2 or less.

^a^ P for trend by the Cochran-Armitage test.

Table 4 shows the ORs of poor intellectual activity for psychosocial conditions, oral health, and dietary variety. After not only adjustment for demographics, lifestyle habits, and physical and mental status (Model 1), but also additional adjustment for psychosocial conditions, oral health measures, and dietary variety (Model 2), lower frequency of social activities, the absence of hobbies and a sense that life is worth living, the lack of regular visits to a dentist, an increase in the number of impaired oral functions, and a lower level of dietary variety were significantly associated with poor intellectual activity. After additional adjustment for IADL (Model 3) or social role (Model 4), these associations were attenuated while remaining significant.

**Table 4:** Odds Ratios of Poor Intellectual Activity (Score <4) for Psychosocial Conditions and Oral Health: Multiple Logistic Regression Analysis.

Variables	Crude OR (95% CI)	Adjusted OR (95% CI)			
		Model 1	Model 2	Model 3	Model 4
**Frequency of participation in social activities (vs. high)**					
Medium	1.51 (1.30–1.76)	1.42 (1.18–1.69)	1.32 (1.10–1.59)	1.30 (1.08–1.56)	1.27 (1.05–1.52)
Low	2.11 (1.80–2.46)	1.86 (1.56–2.20)	1.55 (1.30–1.85)	1.51 (1.26–1.81)	1.41 (1.18–1.69)
Non-participation	3.68 (3.21–4.23)	2.66 (2.27–3.12)	1.90 (1.61–2.24)	1.80 (1.53–2.13)	1.60 (1.35–1.90)
Test for linear trend	p <0.001	p <0.001	p <0.001	p <0.001	p <0.001
**Hobbies and life worth living (*ikigai*) (vs. both hobbies and life worth living (*ikigai*))**					
Hobbies only	2.73 (2.27–3.30)	1.92 (1.57–2.36)	1.68 (1.36–2.07)	1.71 (1.39–2.12)	1.42 (1.15–1.75)
*Ikigai* only	4.45 (3.82–5.17)	3.56 (3.04–4.17)	2.80 (2.38–3.30)	2.66 (2.25–3.14)	2.47 (2.09–2.92)
Neither	6.95 (5.81–8.30)	4.13 (3.39–5.03)	3.13 (2.55–3.84)	2.77 (2.25–3.41)	2.59 (2.11–3.19)
**Dental health behaviors (vs. both regular dental visits and daily brushing)**					
Lack of daily brushing^a^	1.20 (0.89–1.62)	1.16 (0.84–1.59)	1.22 (0.88–1.69)	1.22 (0.88–1.71)	1.24 (0.89–1.74)
Lack of regular dental visits ^b^	1.81 (1.64–2.00)	1.60 (1.45–1.78)	1.39 (1.25–1.55)	1.37 (1.22–1.52)	1.32 (1.18–1.47)
Neither	2.86 (2.35–3.50)	2.08 (1.68–2.59)	1.70 (1.35–2.14)	1.62 (1.28–2.04)	1.61 (1.28–2.03)
**Number of impaired oral functions (i.e., chewing difficulty, choking on tea and soup, and dry mouth) (vs zero)**					
One of three	1.52 (1.36–1.69)	1.26 (1.12–1.42)	1.24 (1.10–1.41)	1.23 (1.09–1.40)	1.22 (1.08–1.38)
Two of three	2.40 (2.11–2.74)	1.62 (1.40–1.87)	1.54 (1.32–1.79)	1.52 (1.30–1.77)	1.50 (1.28–1.75)
All three	3.34 (2.82–3.96)	1.62 (1.33–1.98)	1.61 (1.31–1.98)	1.56 (1.26–1.93)	1.57 (1.27–1.94)
Test for linear trend	p <0.001	p <0.001	p <0.001	p <0.001	p <0.001
**Dietary variety based on the dietary variety score (vs. highest quartile)**					
Second highest quartile	1.42 (1.23–1.63)	1.32 (1.14–1.53)	1.24 (1.07–1.44)	1.23 (1.06–1.44)	1.20 (1.03–1.40)
Second lowest quartile	1.91 (1.67–2.20)	1.74 (1.50–2.01)	1.53 (1.31–1.77)	1.53 (1.31–1.78)	1.43 (1.22–1.66)
Lowest quartile	2.78 (2.45–3.16)	2.33 (2.03–2.67)	1.96 (1.70–2.26)	1.96 (1.69–2.26)	1.86 (1.61–2.15)
Test for linear trend	p <0.001	p <0.001	p <0.001	p <0.001	p <0.001

OR: odds ratio. CI: confidence interval.

^a^ The subjects who had regular dental visits but did not brush daily.

^b^ The subjects who brushed daily but did not have regular dental visits.

Model 1: Adjusted for demographics (age, gender, family structure, pension, and body mass index), lifestyle habits (alcohol and smoking), and physical and mental status (medical history, subjective health status, the number of medications used, cognitive function, depressive symptoms, and falling). Model 2: In addition to model 1, psychosocial conditions, oral health measures, and dietary variety were simultaneously added to the analyses. Model 3: In addition to model 2, instrumental activities of daily living were included. Model 4: In addition to model 2, social role was included.

## Discussion

Less frequency of participation in social activities, not having hobbies, not feeling that life was worth living (no *ikigai*), lack of regular dental visits, more complaints of oral dysfunction, and less dietary variety are associated with poor intellectual activity in nondisabled older Japanese community-dwelling adults. These significant associations persist with adjustment for other independent variables and higher-level functional capacity other than intellectual activity, indicating that psychosocial conditions, oral health, and dietary variety play an independent role in poor intellectual activity. Previous studies have reported that lack of regular visits to a dentist [7], self-perceived chewing disability [8], less dietary variety [9], and lower animal protein intake [15] can increase the risk of decline in higher-level functional capacity in older adults, which supports the results of the present study.

Although several longitudinal studies have reported that deficits in intellectual activity predict a decline in IADL and cognitive function [4–6, 28,29], there are some reports of associations between intellectual activity and other health outcomes, such as depression [30], visual impairment [31], and mortality due to cardiovascular disease and pneumonia [32]. Additionally, intellectual activity was reported to be associated with the frequency of going outdoors [33] and household composition [34]. These findings suggest that intellectual activity may reflect visual function, cardiopulmonary function, and psychosocial status, in addition to IADL and cognitive function.

Regarding possible mechanisms of the observed relationships, we consider the following. For psychosocial conditions, prospective cohort studies of community-dwelling elderly indicated that participation in social activities was protective for the onset of long-term care insurance certification [35] and the incident disability in IADL [21] and ADL [22]. Participation in social activities provides individuals with access to various forms of social support and social network (e.g., access to material resources, or health-relevant information) [36]. This may enhance subjects’ willingness to take interest in health-related information through the mass media including newspapers, books, magazines, and television, which promotes the preservation of intellectual activity. Regarding hobbies and life worth living (*ikigai*), prior studies reported that hobby activities significantly decreased the risk of incident frailty [37], and that the lack of *ikigai* was an independent risk factor for intellectual dysfunction [38], poor self-rated health [39], and cardiovascular risk factors [40]. Because the possession of hobbies and the sense that life is worth living (*ikigai*) reflect an active physiological, behavioral, and psychological profile and a better adaptation to the environment [12,41], the lack of hobbies and a sense that life is worth living may have generally adverse effects on physical and intellectual functioning, which contribute to a negative effect on intellectual activity in elderly people.

For oral health behaviors, they have been shown to be significant contributing factors to promoting oral health status such as oral hygiene and periodontal status [42,43]. Periodontal disease is a common source of chronic infection in humans and is associated with elevated levels of inflammatory markers [44]. Even a low-grade infection in the oral cavity may be associated with a moderate, subclinical systemic inflammatory response, and appropriate treatment reduces levels of inflammatory markers [45]. Chronic inflammation, as measured by serum interleukin 6, is reported to be a risk factor for cognitive decline [46], cardiovascular disease [47], visual degradation [48], poor physical performance [49], and onset of mobility disability [50]. For regular visits to a dentist, two issues may be included; treatment and prevention for dental disease. For subjects having a history of periodontal disease, regular dental attendance is an opportunity to receive suitable dental treatment and prevent adverse health effects of the inflammatory process. Therefore, the lack of regular dental visits may increase the risk for chronic systemic inflammation induced by poor oral health, and lead to a deterioration in cognitive, cardiovascular, visual, and physical functions, resulting in poor intellectual activity. In contrast, for healthy persons without a history of dental disease, there is insufficient evidence to determine the effects of oral prophylaxis [51] (i.e., the removal of plaque, calculus and stain from exposed and unexposed surfaces of the teeth by scaling and polishing as a preventive measure for the control of local irritational factors) [52]. Therefore, it is unclear whether regular dental visits for prevention purpose reduce the risk for future caries and periodontal disease and have an effect on intellectual activity of the healthy elderly. Regarding tooth brushing, our results showed that subjects who brushed at least once a day but did not have regular dental visits were significantly related to poor intellectual activity. Because we categorized brushing frequency as less than once a day or once a day or more and did not assess the quality and quantity of tooth brushing, these subjects may have included a wide variety of people brushing teeth daily. Brushing teeth twice daily is recommended in order to improve plaque control [7,53]. Furthermore, not only the frequency of tooth brushing but also the quality and quantity of tooth brushing are important for oral health maintenance [53,54]. A review shows that meticulous tooth brushing once per day is sufficient to prevent caries and periodontal diseases [54]. Taken together, those subjects may have preponderantly included people brushing teeth insufficiently, and this speculation may be a reasonable explanation for our results.

For oral function, chewing and swallowing disorders are prevalent in the frail elderly, and can cause serious problems such as malnutrition, dehydration, declining quality of diet and aspiration pneumonia [55]. The subjective complaint of oral dryness is also common in older people, and leads to dysphagia, dysgeusia, oral pain, dental caries, oral infection, etc. [56]. Potential pathways have been proposed accordingly. In response to mastication, increased cerebral blood flows and higher oxygen levels were observed within the central nervous system, notably the hippocampus and the prefrontal cortex [57]. Moreover, it is suggested that neuronal signals from teeth influence hippocampus functions [58]. Within the central nervous system, the hippocampus and the prefrontal cortex are considered important for cognitive function [57]. Because intellectual activity, such as completing forms for pensions, reading newspapers, books, or magazines, or being interested in news or programs dealing with health, can be categorized as cognitive stimulating activities [4,5], elderly people who had impairments of oral function may experience a reduction in activation of cognitive function due to mastication, contributing to poor intellectual activity.

For dietary intake, dietary variety has previously been found to be associated with energy intake, meal quality, and biochemical measures of nutritional status in community-dwelling elderly [59,60]. Previous studies of elderly persons have reported that higher dietary intake of protein [61] and vitamin C [62] are associated with higher skeletal muscular strength, lower protein intake is associated with poor cognitive function [63], and low level of blood glucose is associated with poor intellectual activity [14]. Therefore, less dietary variety may hinder elderly people from ensuring an adequate intake of protein, vitamin C, and carbohydrates, leading to decline of physical and cognitive function, resulting in the association between less dietary variety and poor intellectual activity.

This study has some limitations. First, since this is a cross-sectional study, we cannot confirm causal relationships. Longitudinal studies or intervention studies are needed to examine the effects of psychosocial conditions, oral health, and dietary variety on intellectual activity. Second, psychosocial conditions, oral health, dietary variety, and intellectual activity were assessed by self-report. Therefore, associations among the study variables may be overestimated due to a common response style while several studies have shown that these influences are not as much as that one could expect [64]. Third, it has been pointed out that the adjusted OR from the logistic regression may exaggerate a risk association or a treatment effect when the incidence of an outcome of interest is common in the study population (more than 10 percent) [65,66]. It should be paid attention to insomuch that the limitation that our findings may overestimate the association between psychosocial conditions, oral health, dietary variety, and intellectual activity, because our study population has a high prevalence of such outcome. Fourth, our results are biased by the exclusion of subjects who could not obtain the required valid data or did not return to the questionnaire. Proportions of individuals aged 75 years and older were greater in individuals with invalid data than in those with valid data (53.6% vs 37.2%). Although we do not have any information about the non-responders, they might have suffered from poor psychosocial conditions, poor oral health, poor dietary intake, or poor functional capacity, which could have hindered the ability to participate in this study. We speculate that the high-risk population for disability was excluded in this study. This may have resulted in an underestimation of the association between psychosocial conditions, oral health, dietary variety, and intellectual activity. Fifth, we cannot rule out the possibility of residual confounding as there could have been several unmeasured parameters such as societal or lifestyle factors that could have influence on intellectual activity. For example, we lacked information on educational background. Because prior studies have suggested that low educational attainment is associated with higher-level functional capacity [11, 14] as well as psychosocial conditions [67,68], oral health [69], and dietary intake [15], additional adjustment for educational level might have changed the observed associations. Finally, our study participants were all from one medium-sized municipality in Japan, and our analytical subjects were independent in terms of their ADL. Therefore, generalization of the findings should be done with caution.

Despite these limitations, to the best of our knowledge, this is the first study to investigate the association of psychosocial conditions, oral health, and dietary intake with intellectual activity in the community-dwelling elderly. Our results indicate the possibility that support for community-dwelling non-disabled elderly that encourages participation in social activities, possession of hobbies and life worth living (*ikigai*), regular visits to the dentist, treatment and rehabilitation of oral dysfunction, and intake of a variety of foods is effective for maintaining intellectual activity ability.

## Supporting Information

S1 Table The Tokyo Metropolitan Institute of Gerontology Index of Competence (TMIG-IC) for Assessing Higher-level Functional Capacity in Older Adults. (PDF)
