# Supplementary material for: Association of Psychosocial Conditions, Oral Health, and Dietary Variety with Intellectual Activity in Older Community-Dwelling Japanese Adults
Source: PLoS One. 2015 Sep 11;10(9):e0137656. doi: 10.1371/journal.pone.0137656 (PMC4567331; doi:10.1371/journal.pone.0137656)
Supplement: S1 Table — (PDF) [file pone.0137656.s001.pdf]

S1 Table. The Tokyo Metropolitan Institute of Gerontology Index of Competence (TMIG-IC) for Assessing Higher-level Functional Capacity in Older Adults

Sub-scales		Questionnaires	
Instrumental Activities of Daily Living			
1	Can you use public transportation (bus or train) by yourself ?	1. Yes	0. No
2	Are you able to shop for daily necessities ?	1. Yes	0. No
3	Are you able to prepare meals by yourself ?	1. Yes	0. No
4	Are you able to pay bills ?	1. Yes	0. No
5	Can you handle your own banking ?	1. Yes	0. No
Intellectual Activity			
6	Are you able to fill out forms for your pension ?	1. Yes	0. No
7	Do you read newspapers ?	1. Yes	0. No
8	Do you read books or magazines ?	1. Yes	0. No
9	Are you interested in news stories or programs dealing with health ?	1. Yes	0. No
Social Role			
10	Do you visit the homes of friends ?	1. Yes	0. No
11	Are you sometimes called on for advice ?	1. Yes	0. No
12	Are you able to visit sick friends ?	1. Yes	0. No
13	Do you sometimes initiate conversations with young people ?	1. Yes	0. No
